# The cytoplasmic domain of the pseudoprotease iRhom2 mediates distinct signaling mechanisms to control activation of the cell surface protease ADAM17

**DOI:** 10.1016/j.jbc.2025.110643

**Published:** 2025-08-28

**Authors:** Fangfang Lu, Marjorie Fournier, Matthew Freeman

**Affiliations:** 1 Sir William Dunn School of Pathology, University of Oxford, Oxford, UK; 2 Advanced Proteomics Facility, Department of Biochemistry, University of Oxford, Oxford, UK

**Keywords:** membrane protein, iRhom2, ADAM17, RSK, FRMD8, KRAS, cell surface, shedding

## Abstract

ADAM17 is a cell surface protease that controls the release of the ectodomains of signaling proteins, including epidermal growth factor receptor ligands and the primary inflammatory cytokine tumor necrosis factor. Reflecting this important role in signaling, dysregulated ADAM17 activity is linked to many human diseases, including immunodeficiency, inflammatory bowel disease, rheumatic arthritis, cancer, and Alzheimer's disease. iRhom2, a pseudoprotease of the rhomboid-like superfamily, has evolved to be a multifunctional regulatory cofactor of ADAM17. Recent structural and functional work has begun to reveal how the iRhom2 transmembrane and extracellular domains act to control ADAM17 activity. The cytoplasmic domain, however, remains less explored. Here, using a combination of proteomic, genetic, and biochemical approaches, we report three distinct mechanisms by which the cytoplasmic domain of iRhom2 contributes to ADAM17 regulation. First, upon oncogenic KRAS signaling, the serine/threonine kinase RSK2 is recruited to the iRhom2 cytoplasmic N terminus and coordinates with phosphorylated ERK kinase to activate the iRhom2–ADAM17 sheddase complex. Second, we show that iRhom2 may have an inhibitory function on ADAM17 at the cell surface: stabilizing iRhom2 at the cell surface by overexpressing iRhom2's cytoplasmic binding partner, FRMD8, inhibits PMA–stimulated ADAM17 activity. Third, we have identified a previously undefined motif (RKR) in the iRhom2 cytoplasmic domain that represses unstimulated ADAM17 activity. Overall, these findings reveal the complex regulatory system by which the iRhom2 cytoplasmic tail transduces cellular signals to regulate ADAM17 activation, potentially paving the way toward understanding and possibly manipulating the iRhom2–ADAM17 complex in health and disease.

Many important signals between cells are triggered by proteolytic shedding of extracellular domains of transmembrane proteins (1). One of the primary “sheddase” enzymes responsible for this release of signals from the cell surface is the metalloprotease a disintegrin and metalloproteinase 17 (ADAM17) (also known as tumor necrosis factor-α-converting enzyme, TACE). ADAM17, like many other sheddases, has a transmembrane domain (TMD), which anchors the metalloprotease domain to the cell surface (Fig. 1*A*); it has many substrates, including, most prominently, the central proinflammatory cytokine tumor necrosis factor and growth factors of the epidermal growth factor (EGF) family (2). Given the central role of ADAM17 in triggering inflammatory and growth factor signaling (3, 4, 5, 6), its activity, cellular localization, and substrate selectivity must be tightly regulated. Over the last decade, it has become clear that a substantial element of that control is provided by the iRhom proteins, which have evolved as regulatory cofactors of ADAM17 (7, 8, 9, 10) (Fig. 1*A*). The iRhoms are pseudoproteases from the rhomboid-like superfamily, an evolutionarily conserved class of polytopic membrane proteins involved in a wide range of biological processes, including, for example, signaling, endoplasmic reticulum (ER)–associated degradation, mitochondrial function, and parasite infection (11, 12). There are two iRhoms in mammalian cells, iRhom1 and iRhom2, both of which can regulate ADAM17 but which have different tissue distributions, as well as subtle functional distinctions (13).

**Figure 1:**
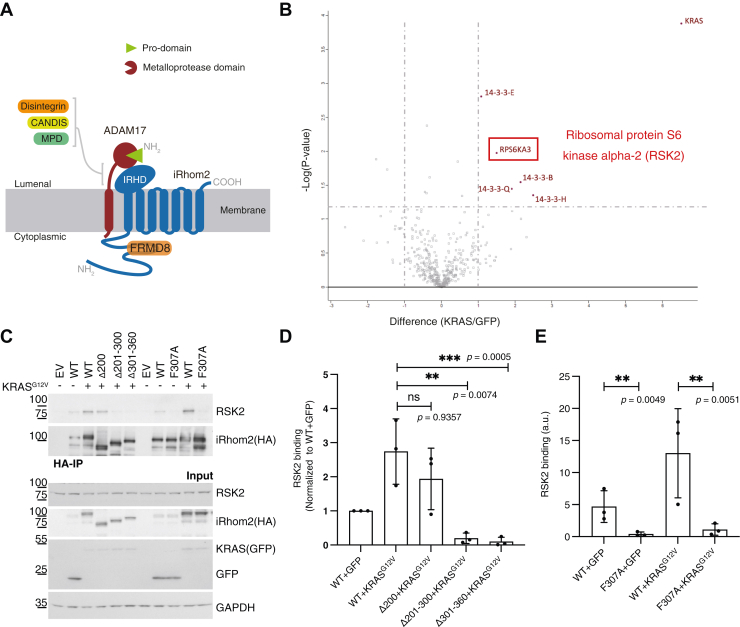
**RSK2 is recruited to the iRhom2 cytoplasmic domain upon oncogenic KRAS expression.***A,* a schematic of the iRhom2–ADAM17 sheddase complex. ADAM17 is a single-pass membrane protein (*red*) with a prodomain (*green*), a metalloprotease domain, a disintegrin domain, a conserved ADAM17 dynamic interaction sequence (CANDIS) domain, an membrane proximal domain (MPD) domain, a transmembrane domain (TMD), and a cytoplasmic domain. iRhom2 is a membrane protein (*blue*), with seven TMDs, a conserved lumenal domain, called the iRhom homology domain (IRHD), and an extended cytoplasmic domain. The cytosolic stabilizing factor, FERM domain containing 8 (FRMD8) (*orange*), interacts with iRhom2 cytoplasmic domain. *B,* a scatter plot showing the proteins recruited to WT iRhom2 (HA-tagged) upon oncogenic KRAS expression compared to the GFP control. Ribosomal protein S6 kinase alpha-3 (RPS6KA3), also called RSK2, is highlighted in a *red box*. The statistical analysis was performed on NSAF values from three independent experiments using a *t* test. Proteins with a minimum fold change of two between each condition and a *p* value cutoff of 0.05 are highlighted on the plot. *C,* iRhom1/2 double KO (DKO) cells were transfected with either empty vector (EV), WT iRhom2, or different iRhom2 variants together with GFP-tagged KRAS^G12V^ or GFP as control. An HA-based pulldown assay was performed to assay the interaction between iRhom2 and endogenous RSK2. HA-immunoprecipitants and cell lysates were blotted for endogenous RSK2, iRhom2 (HA), GFP, KRAS (GFP), and GAPDH as a loading control. *D* and *E,* quantification from three independent experiments of Figure 1C. Images of Western blots were quantified using Fiji2. RSK2 binding of each condition is normalized to the WT + GFP condition for comparison in *D*. Log_10_-transformed data were statistically analyzed using ordinary one-way ANOVA, followed by Dunnett's multiple comparisons test for *D* and Šídák's multiple comparisons test for *E* for pairwise comparisons as indicated. ns = not significant; ∗ *p* < 0.05; ∗∗ *p* < 0.01; ∗∗∗ *p* < 0.001. Adjusted *p* values are shown. Error bars represent the SD. ADAM17, a disintegrin and metalloproteinase 17; NSAF, normalized spectral abundance.

We now understand some important aspects of the multiple ways in which ADAM17 is controlled by iRhoms. First, the primary interaction between iRhoms and ADAM17 is mediated by their respective TMDs, which is a hallmark of rhomboid-like proteins. The genetic and biochemical evidence for this (14, 15) has recently been confirmed and reinforced by the solution of the ADAM17–iRhom2 protein complex structure (16). In the absence of the TMD interaction, ADAM17 cannot leave the ER, and therefore, it neither traffics to the cell surface nor is matured by furin-dependent removal of the inhibitory prodomain (7, 8, 13, 17). Second, iRhoms have also been implicated in modulating ADAM17 substrate selectivity (18, 19). Third, the recent structure revealed the regulatory significance of iRhom2's highly conserved lumenal loop domain, the iRhom homology domain (IRHD), located between the first two transmembrane helices (20). Interactions between this IRHD and the extracellular parts of ADAM17 control the activation of ADAM17 in the late secretory pathway by regulating both the release from the complex of the furin-cleaved inhibitory prodomain and the mobility of the catalytic domain (16).

Despite these recent advances in understanding how iRhoms have been repurposed through evolution to become multifunctional regulators of ADAM17, we know much less about the importance of the iRhom cytoplasmic domain. The extended cytoplasmic amino terminus (over 400 amino acids long) of iRhom2 is predicted to be largely intrinsically disordered. Consistent with this, the cytoplasmic domain was not seen in the cryo-EM structure of the iRhom2–ADAM17 complex (16), as cryo-EM is unable to resolve unstructured, and therefore highly mobile, domains. Nevertheless, functional studies have shown that phosphorylation of the iRhom2 cytoplasmic domain and subsequent recruitment of 14-3-3 proteins are essential for the stimulated activity of ADAM17 (9, 10, 21). The iRhom2 N terminus also binds the cytosolic protein, FERM (Four-point-one, Ezrin, Radixin, Moesin) domain containing 8 (FRMD8, also called iTAP) (Fig. 1*A*), which plays an essential role in stabilizing the mature ADAM17–iRhom complex at the cell surface (22, 23). Finally, the significance of the iRhom2 cytoplasmic domain is highlighted by human genetics: point mutations in a very small region within this domain cause a human cancer syndrome called tylosis with esophageal cancer, also known as Howel–Evans syndrome (24, 25, 26, 27). These disease mutations potentially mediate a positive-feedback loop in oncogenic KRAS signaling (21) and have recently been shown to be part of a noncanonical 14-3-3 binding site (28). In summary, while there are ample functional data pointing to the regulatory significance of the iRhom2 cytoplasmic N terminus, our understanding of its mechanistic significance is far from comprehensive.

Here, we reveal further elements of the regulatory significance of the iRhom2 cytoplasmic domain. First, using mass spectrometry (MS) to explore the interactome, we found that ribosomal protein S6 kinase alpha-3 (RPS6KA3, also called RSK2) is recruited to the iRhom2 cytoplasmic domain upon oncogenic KRAS signaling. Second, we explored the relationship between iRhom2 stability at the cell surface and ADAM17 stimulation, finding a significant decrease in plasma membrane iRhom2 level when ADAM17 is activated, a process that can be modulated by the iRhom2 cytoplasmic interactor, FRMD8. Third, we have identified a previously unknown motif (RKR) in the iRhom2 cytoplasmic domain, mutation of which leads to elevated unstimulated ADAM17 activity. These three newly identified mechanisms mediated by the iRhom2 cytoplasmic domain shed light on the multiple modes by which it has evolved as a dedicated regulator of ADAM17. Overall, this work reinforces our emerging understanding of iRhom2 as a modular and multifunctional regulator of ADAM17 shedding activity, with different parts of the protein (the TMDs, the extracellular IRHD, and the extended cytoplasmic domain) all participating in the tight control of ADAM17-dependent inflammatory and growth factor signaling.

## Results

### RSK2 is recruited to the iRhom2 cytoplasmic domain upon oncogenic KRAS expression

We have reported that oncogenic KRAS signaling activates the sheddase activity of ADAM17 and that the phosphorylation of the iRhom2 N terminus is key to this process (21). To identify the machinery of this oncogenic activation, we used an MS experiment to identify proteins that bind to iRhom2 in the presence or absence of the oncogenic KRAS G12V mutant (Fig. 1*B*). Consistent with earlier reports of activated shedding (9, 10, 21), multiple isoforms of 14-3-3 proteins were recruited to iRhom2 upon oncogenic KRAS expression. In the same experiment, the serine/threonine kinase RPS6KA3 (also called RSK2) was recruited to iRhom2 in the presence of oncogenic KRAS. The interaction between iRhom2 and endogenous RSK2 was validated by Western blot (Fig. 1*C*). A series of iRhom2 N-terminal truncations revealed that amino acids 300 to 360 in the iRhom2 cytoplasmic domain are responsible for binding RSK2 (Fig. 1*C*, quantification in Fig. 1*D*). This region includes a conserved VF amino acid pair, which was recently identified to be a major determinant of RSK2 binding to its substrate (29). Indeed, we found that a single mutation of F307A in the iRhom2 cytoplasmic domain completely abolished its interaction with RSK2 (Fig. 1*C*, quantification in Fig. 1*E*). It is notable that the VF motif is conserved between human iRhom1 and iRhom2, suggesting that RSK2 may regulate both.

### RSK2 is required for oncogenic KRAS–induced ADAM17 activation

**Figure 2:**
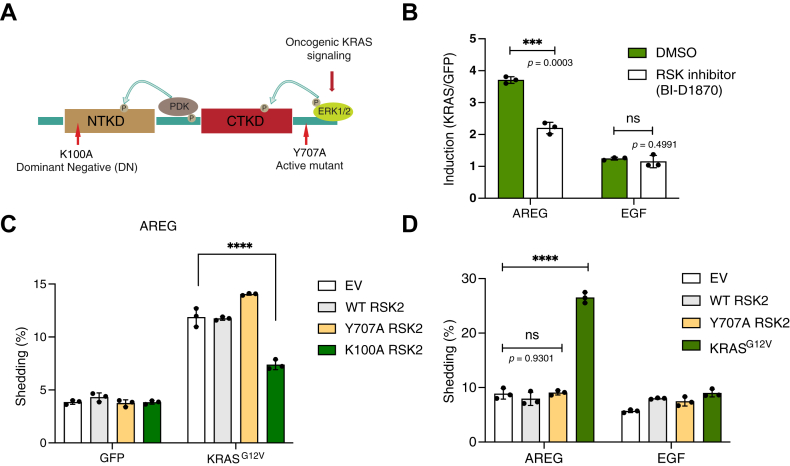
**RSK2 is required for oncogenic KRAS signaling–induced ADAM17 activation.***A,* a schematic of the RSK2 activation mechanism and mutations, which render RSK2 constitutively active (Y707A) or dominant negative (DN, K100A). *B,* AP-shedding assay of cells expressing GFP or oncogenic KRAS (G12V) together with ADAM17 substrate AREG or ADAM10 substrate EGF. Induction of ADAM17 activity by oncogenic KRAS: divide the shedding percentage of KRAS condition by the shedding percentage of GFP condition (KRAS/GFP). Cells were treated with 10 μM BI-D1870 or DMSO as a control. *C,* AP-shedding assay of cells expressing GFP or oncogenic KRAS (G12V) when different variants of RSK2 were used. *D,* AP-shedding assay of cells expressing WT, active Y707A RSK2, or oncogenic G12V KRAS. For *B*–*D*, log_10_-transformed data were statistically analyzed using ordinary one-way ANOVA, followed by Šídák's multiple comparisons test for pairwise comparisons as indicated. ns = not significant; ∗∗∗ *p* < 0.001; ∗∗∗∗ *p* < 0.0001. Adjusted *p* values are shown. Error bars represent the SD. n = 3 transfectants, medium collected and assayed separately. Quantification of the three independent experiments is shown in Figure S1, *A*, *B* and *D*. ADAM10/17, A disintegrin and metalloproteinase 10/17; AP, alkaline phosphatase; AREG, amphiregulin; CTKD, C-terminal kinase domain; DMSO, dimethyl sulfoxide; EGF, epidermal growth factor; EV, empty vector; NTKD, N-terminal kinase domain.

RSK2 belongs to the p90 ribosomal S6 kinase family, which works downstream of extracellular signal–regulated kinase (ERK) (Fig. 2*A*) and is therefore an important component of the KRAS signaling pathway (30, 31, 32, 33, 34). We therefore asked if RSK2 is required for oncogenic KRAS–induced ADAM17 activation by using a highly specific RSK inhibitor, BI-D1870 (35). In a well-established alkaline phosphatase (AP)–shedding assay that measures ADAM17 activity, BI-D1870 significantly reduced the shedding of the ADAM17 substrate amphiregulin (AREG) induced by oncogenic KRAS (Figs. 2*B* and S1*A*), implying that RSK2 does indeed participate in the stimulation of ADAM17 by oncogenic KRAS. The inhibitor had no effect on the shedding of EGF by ADAM10 (Figs. 2*B* and S1*A*), a metalloprotease related to ADAM17 but which is not regulated by iRhoms (10), demonstrating the specificity of the RSK2 contribution to iRhom2–ADAM17 activation. A dominant negative RSK2 mutant provides another independent line of evidence for the role of RSK2: expression of the K100A mutant RSK2 (36, 37) in cells expressing oncogenic KRAS significantly reduced AREG shedding (Figs. 2*C* and S1*B*) without affecting EGF shedding (Fig. S1*B*). Consistent with this, the F307A iRhom2 also showed reduced ability to support oncogenic KRAS–induced ADAM17 activity (Fig. S1*C*). Thus, both genetic and pharmacological experiments support an integral role for RSK2 in oncogenic KRAS–induced ADAM17 activation.

It has been established that ERK is involved in the activation of the iRhom2–ADAM17 sheddase complex (9, 10). Since RSK2 can work downstream of ERK (38), it is attractive to hypothesize that ERK and RSK2 might work in a simple linear cascade to phosphorylate and activate iRhom2. However, we found that a constitutively active mutant of RSK2 (Y707A) (36, 37, 39) was unable to drive ADAM17 activity on its own (Fig. 2*D* and S2*D*), implying that activated RSK2 alone is insufficient to drive ADAM17 activation. Our data therefore demonstrate that RSK2 is recruited to the iRhom2 cytoplasmic domain upon oncogenic KRAS overexpression and that it is needed for efficient activation of ADAM17, but that ERK and RSK2 participate in an interdependent network of signaling rather than a simple linear module.

### iRhom2 cell surface level decreases significantly upon phorbol 12-myristate 13-acetate treatment

Having identified RSK2 as a contributor to ADAM17 activation driven by oncogenic KRAS signaling, we next sought to investigate the activation process in more detail, focusing specifically on iRhom2 and ADAM17. Given that ADAM17 functions as a sheddase, releasing substrate ectodomains into the extracellular spaces, its shedding activity is generally assumed to occur at or near the plasma membrane. However, the dynamics of iRhom2 and ADAM17 at the cell surface during this activation process remain largely unexplored.

**Figure 3:**
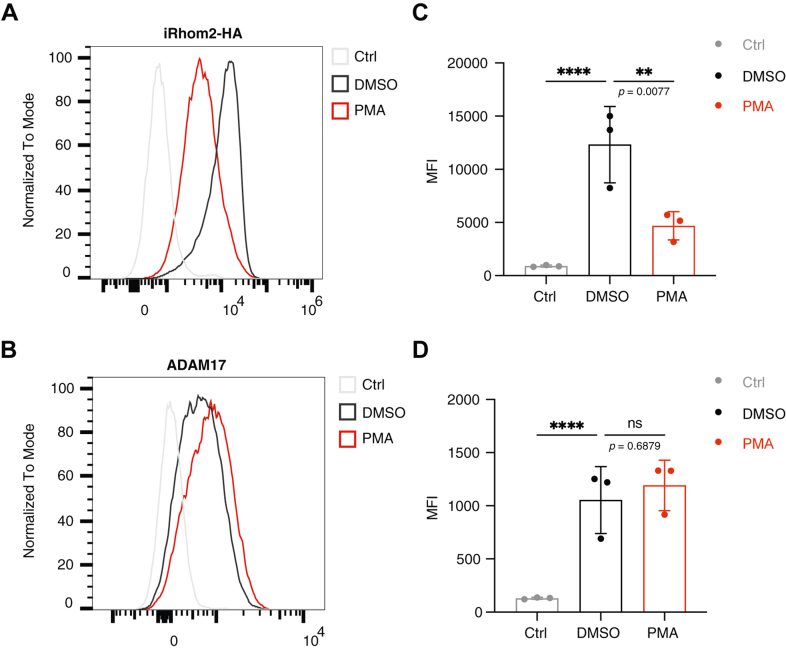
**iRhom2 showed a drastic decrease in its cell surface level upon PMA treatment.***A* and *B,* flow cytometry analysis of cell surface iRhom2 and ADAM17. iRhom1/2 DKO cells stably re-expressing iRhom2 (HA tagged) were treated with PMA (200 nM, 15 min) or DMSO as a control. Cells were stained with an anti-HA-tag antibody or anti-ADAM17 ectodomain antibody, followed by fluorescent secondary antibodies. Negative control (Ctrl) showed cells only stained with fluorescent secondary antibodies. Histograms show live cells from one representative experiment. *C* and *D,* median fluorescence intensity (MFI) of iRhom2 (HA tagged) and ADAM17 proteins at the cell surface when cells were treated with DMSO and PMA. Quantification was from three independent experiments. Log_10_-transformed data were statistically analyzed using ordinary one-way ANOVA, followed by Dunnett's multiple comparisons test against the DMSO condition. ns = not significant; ∗∗ *p* < 0.01, ∗∗∗∗ *p* < 0.0001. Adjusted *p* values are shown. Error bars represent the SD. ADAM17, a disintegrin and metalloproteinase 17; DKO, double KO; DMSO, dimethyl sulfoxide; PMA, phorbol 12-myristate 13-acetate.

It has been reported that phorbol 12-myristate 13-acetate (PMA) treatment significantly reduces the strength of the interaction between mouse iRhom2 and ADAM17 at the cell surface (9, 10), indicating disassembly, or at least weakening, of the cell surface iRhom2–ADAM17 complex upon activation. Using cell surface labeling followed by flow cytometry, we measured the surface level of human iRhom2 (HA-tagged) and ADAM17 (detected using an antibody against its ectodomain). Strikingly, PMA treatment led to a substantial decrease in cell surface iRhom2, whereas cell surface ADAM17 remained largely unaffected (Fig. 3, *A* and *B*, quantifications in Fig. 3, *C* and *D*). This suggests that iRhom2 and ADAM17 might experience different fates and follow different trafficking routes during and after PMA-induced activation. The observed downregulation of cell surface iRhom2 in correlation with PMA-induced ADAM17 activation also hints that iRhom2 might play an inhibitory role in regulating ADAM17 activity at the plasma membrane.

### Stabilizing iRhom2 at the cell surface by overexpressing iRhom2's cytoplasmic binder FRMD8 inhibits PMA-stimulated ADAM17 activation

The observation that iRhom2 level at the cell surface significantly drops upon ADAM17 stimulation and activation suggested that stabilizing iRhom2 at the cell surface might hinder ADAM17 activity. We took advantage of the cytosolic interactor of iRhom2, FRMD8, to manipulate the cell surface levels of iRhom2 in cells. FRMD8 binds the iRhom2 cytoplasmic domain and acts as a stabilizing factor for the iRhom2–ADAM17 sheddase complex at the plasma membrane (Fig. 1*A*) (22, 23).

**Figure 4:**
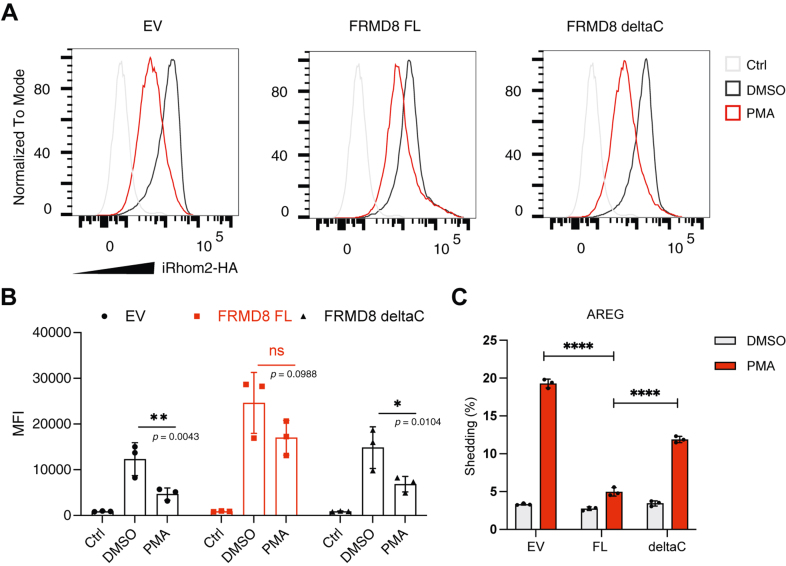
**Overexpression of full-length (FL) FRMD8, but not the C-terminal deleted mutant, suppresses the PMA-stimulated ADAM17 shedding activity.***A,* flow cytometry analysis of cell surface iRhom2. iRhom1/2 DKO cells stably re-expressing iRhom2 (HA tagged) were transfected with either EV (empty vector), FL, or deltaC (C-terminal deleted) FRMD8. Cells were treated with PMA (200 nM, 15 min) or DMSO as control. Cells were stained with an anti-HA-tag antibody, followed by a fluorescent secondary antibody. Negative control (Ctrl) showed untransfected cells only stained with a fluorescent secondary antibody. Histograms represent live cells. *B,* median fluorescence intensity (MFI) of iRhom2 (HA tagged) at the cell surface when cells were treated with DMSO and PMA. Quantification was from three independent experiments. Log_10_-transformed data were statistically analyzed using ordinary one-way ANOVA, followed by Šídák's multiple comparisons test for pairwise comparisons as indicated. ns = not significant; ∗ *p* < 0.05, ∗∗ *p* < 0.01, ∗∗∗∗ *p* < 0.0001. Adjusted *p* values are shown. Error bars represent the SD. *C,* AP-shedding assay of ADAM17 substrate AREG. iRhom1/2 DKO HEK293T cells stably re-expressing iRhom2 were transfected with either EV, FL FRMD8, or deltaC (C-terminal truncated FRMD8) together with AP–AREG. Cells were stimulated with 200 nM PMA or DMSO as a control, and the medium was collected for 1 h. Log_10_-transformed data were statistically analyzed using ordinary one-way ANOVA, followed by Šídák's multiple comparisons test for pairwise comparisons as indicated. ∗∗∗∗ *p* < 0.0001. n = 3 transfectants, medium collected separately. Error bars represent the SD. Quantification of the three independent experiments is shown in Figure S2*A*. ADAM17, a disintegrin and metalloproteinase 17; AP, alkaline phosphatase; AREG, amphiregulin; DKO, double KO; DMSO, dimethyl sulfoxide; FRMD8, FERM domain containing 8; PMA, phorbol 12-myristate 13-acetate.

As expected, overexpression of the full-length FRMD8 (FL FRMD8) significantly increased the cell surface protein level of iRhom2 compared with the empty vector control (Fig. 4*A*). Interestingly, while PMA stimulation significantly reduced iRhom2 cell surface level, cells overexpressing an FL FRMD8 retained a significant amount of iRhom2 at the cell surface after PMA stimulation (Fig. 4*A*, see quantification in Fig. 4*B*). In accordance with our hypothesis that stabilizing iRhom2 at the cell surface inhibits stimulated ADAM17 activity, when FL FRMD8 was overexpressed, the PMA-stimulated shedding activity of ADAM17 was significantly reduced (Figs. 4*C*, and S2*A*). The ADAM10-mediated shedding activity (an iRhom2-independent control) was unaffected (Fig. S2*B*), confirming the specificity of this phenomenon.

FRMD8 belongs to the family of FERM domain–containing proteins (40); the C terminus of these proteins often harbors the regulatory functions, including, for example, PIP_2_ binding and F-actin binding (41, 42). Indeed, the C terminus of FRMD8 was observed to play a role in regulating the stability of the iRhom2–ADAM17 complex (43). When the C terminus of FRMD8 was deleted (deltaC FRMD8, amino acids 381–464 deleted), the protein showed impaired ability to stabilize iRhom2 at the cell surface (Fig. 4A); moreover, PMA-stimulated ADAM17 shedding activity was much less affected by this deletion mutant than by FL FRMD8 (Figs. 4*C* and S2*A*). Both these results indicate a negative correlation between iRhom2 cell surface levels and ADAM17 activation: a high iRhom2 cell surface level correlates with a low ADAM17 activity; conversely, PMA-stimulated ADAM17 activation leads to a reduced iRhom2 cell surface level.

### A novel polybasic motif in the iRhom2 cytoplasmic domain regulates unstimulated ADAM17 activation

The two mechanisms we have reported previously, the role of RSK2 in mediating oncogenic activation of ADAM17 and the relationship between cell surface iRhom2 levels and ADAM17 activity, both concern the regulation of stimulated ADAM17 activity. We now describe a distinct, cytoplasmic mode of iRhom2-mediated regulation that controls basal, unstimulated ADAM17 activity.

**Figure 5:**
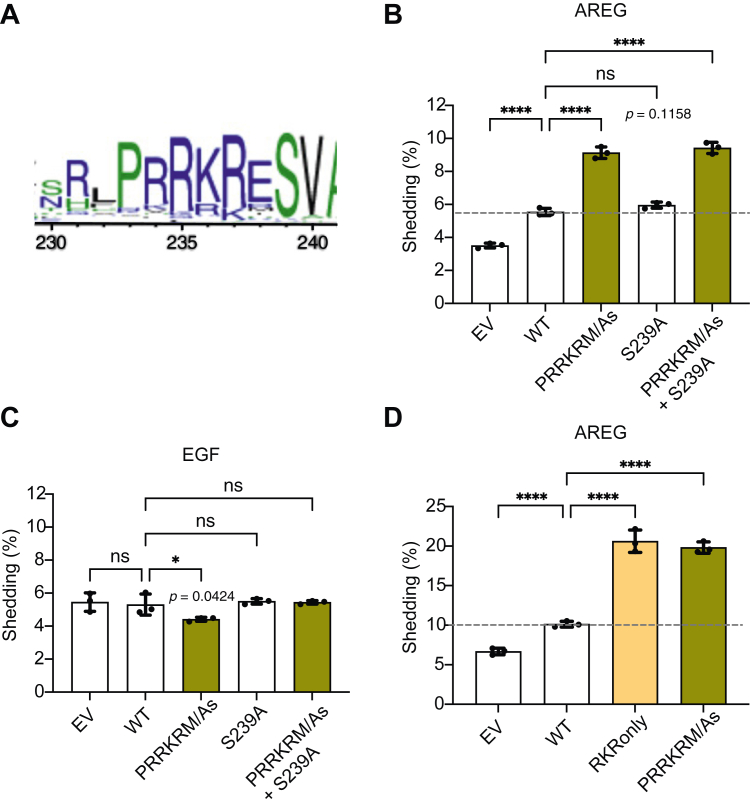
**Mutating the RKR motif in the iRhom2 cytoplasmic domain leads to elevated constitutive shedding acidity of ADAM17.***A,* residue conservation analysis produced by GREMLIN at OPENSEQ.org. The graphical representation of amino acid multiple sequence alignment was generated by WebLogo. Color shows hydrophobicity (*blue*: hydrophilic, *green*: neutral, and *black*: hydrophobic). The overall height of the stack is used to indicate the sequence conservation at that position, and the height of individual symbols within the stack indicates the relative frequency of each amino acid at that position. *B*–*D,* AP-shedding assay of iRhom1/2 DKO HEK293T cells expressing EV (empty vector), WT, or different mutant iRhom2. Cells were not stimulated, and medium was collected overnight. AREG was used as an ADAM17 substrate, and EGF (ADAM10 substrate) was used as a control. For *B*–*D,* log_10_-transformed data were statistically analyzed using ordinary one-way ANOVA, followed by Dunnett's multiple comparisons test against the WT condition. ns = not significant; ∗ *p* < 0.05; ∗∗∗∗ *p* < 0.0001. Adjusted *p* values are shown. n = 3 transfectants, medium collected and assayed separately. Error bars represent the SD. Repeats of the experiments of Figure 5, *B* and *D* are shown in Figure S3. ADAM17, a disintegrin and metalloproteinase 17; AP, alkaline phosphatase; AREG, amphiregulin; DKO, double KO; EGF, epidermal growth factor; HEK293T, human embryonic kidney 293T cell line.

Because the cytoplasmic tail of iRhom2 is predicted to be intrinsically disordered, with few expected secondary structures, we postulated that protein interactions might be mediated by short linear motifs (44, 45, 46). Using the Eukaryotic Linear Motif Prediction tool (47), we identified multiple already known protein interaction sites, including 14-3-3 binding and ERK docking sites (9, 10). This approach also highlighted a previously unrecognized polybasic residue motif, PRRKRM (iRhom2 isoform1 residues 233–238), where the sequence RRKRM (234–238) was predicted to be an ER retrieval and retention signal (48, 49). In addition, conservation analysis *via* multiple sequence alignment using the GREMLIN algorithm at OPENSEQ.org showed that the overlapping sequence PRRKR (233–237) is highly conserved across species (Fig. 5*A*). Combining these overlapping sequences of potential interest, we generated a mutant iRhom2, in which all the amino acids in this PRRKRM sequence were mutated into alanine (PRRKRM/As). We asked whether this mutant affected iRhom2–ADAM17-dependent shedding. Strikingly, mutation of this motif in iRhom2 activated ADAM17, resulting in elevated basal AREG release even in the absence of stimulating signals (Fig. 5*B*). As a negative control, we showed that shedding by ADAM10 was unaffected (Fig. 5*C*). Using further mutagenesis, we narrowed down the essential residues RKR (235–237) as the core functional motif in this sequence. The RKR-only mutant, in which all three residues were replaced by alanine, had a similar effect to the PRRKRM/As mutant in upregulating constitutive ADAM17 shedding activity (Fig. 5*D*).

Short linear motif binding is often regulated by nearby phosphorylation (50), and we noted the presence of a highly conserved serine at position 239 in iRhom2. However, mutating this residue to alanine (S239A), to prevent its potential phosphorylation, did not affect ADAM17 shedding activity and had no additive effect on the PRRKRM/As mutant (Fig. 5*B*). We therefore conclude that S239 does not influence the role of the RKR motif.

In summary, we have identified the RKR (235–237) sequence in the iRhom2 cytoplasmic domain as a novel motif that regulates the activity of ADAM17: its mutation leads to excess basal shedding of ADAM17 substrates.

### Abnormal ADAM17 activation caused by iRhom2 RKR mutation leads to mature ADAM17 degradation

**Figure 6:**
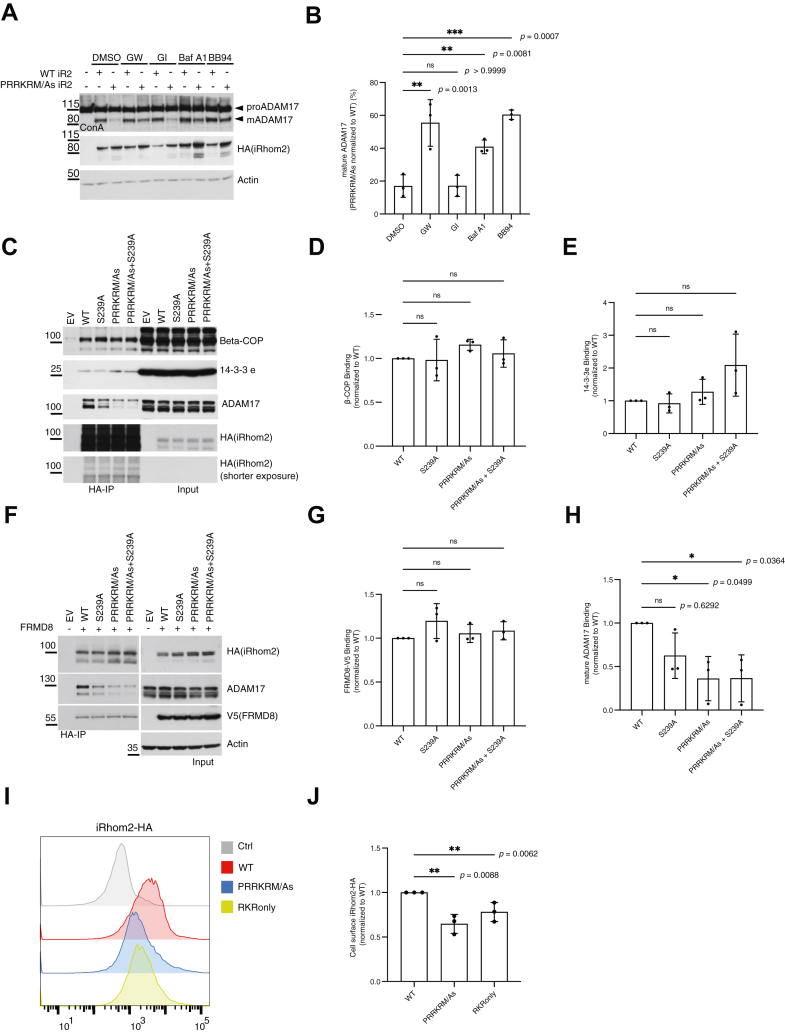
**Mutating the PRRKRM motif in the iRhom2 cytoplasmic domain weakens iRhom2–ADAM17 interaction and leads to mature ADAM17 degradation.***A,* iRhom1/2 DKO HEK293T cells were transfected with WT or PRRKRM/As mutant iRhom2. Concanavalin A (ConA) enrichment and cell lysates were probed for ADAM17, HA (iRhom2) and ADAM17 and actin as a control. Cells were treated with 2 μM GW, 2 μM GI, 10 μM BB94, or 100 mM lysosomal inhibitor bafilomycin A1 (Baf A1) for 12 h before cells were harvested. *B,* quantification from three independent experiments of Figure 6A. The levels of mature ADAM17 in cells expressing the PRRKRM/As mutant iRhom2 were normalized to those in cells expressing WT iRhom2 for each drug treatment. Log_10_-transformed data were statistically analyzed using ordinary one-way ANOVA, followed by Dunnett's multiple comparisons test against the DMSO condition. ns = not significant; ∗∗ *p* < 0.01; ∗∗∗ *p* < 0.001. Adjusted *p* values are shown. Error bars represent SD. *C,* iRhom1/2 DKO HEK293T cells were transfected with empty vector (EV) control, WT iRhom2, PRRKRM/As, or RKR-only mutant iRhom2. HA-based immunoprecipitates and lysates were blotted for Beta-COP, 14-3-3 epsilon (e), ADAM17, and HA (iRhom2). *D, E, H,* quantifications from three independent experiments of Figure 6*C*. Levels of β-COP, 14-3-3 epsilon (e), and mature ADAM17 detected in HA-IP samples from cells expressing WT or mutant iRhom2 were normalized to the corresponding levels in cells expressing WT iRhom2. *F,* iRhom1/2 DKO HEK293T cells were transfected with EV control, WT iRhom2, PRRKRM/As, or RKR-only mutant iRhom2 together with V5-tagged FRMD8. HA-based immunoprecipitates and lysates were blotted for ADAM17, HA (iRhom2), V5 (FRMD8), and actin. *G,* quantification from three independent experiments of Figure 6*F*. Levels of FRMD8 (V5 tagged) protein detected in the HA-IP samples from cells expressing WT or mutant iRhom2 were normalized to the corresponding levels in cells expressing WT iRhom2. For *D, E, H,* and *G,* log_10_-transformed data were statistically analyzed using the Kruskal–Wallis test, followed by Dunnett's multiple comparisons test against the WT condition. ns = not significant. ∗ *p* < 0.05. Adjusted *p* values are shown. Error bars represent the SD. *I,* flow cytometry analysis of cell surface iRhom2. iRhom1/2 DKO HEK293T cells were transfected with empty vector (EV) control, WT iRhom2, PRRKRM/As, or RKR-only mutant iRhom2 and stained with an anti-HA tag antibody followed by a fluorescent secondary antibody. Negative control (Ctrl) showed untransfected cells only stained with a fluorescent secondary antibody. Histograms represent live cells. *J,* quantification of three independent experiments from Figure 6*I*. Median fluorescence intensity (MFI) of iRhom2 (HA-tagged) proteins at the cell surface of mutant iRhom2 was normalized to WT iRhom2. Log_10_-transformed data were statistically analyzed using a one-sample *t* test. ns = not significant; ∗∗ *p* < 0.01. *p* Values are shown. Error bars represent the SD. ADAM17, a disintegrin and metalloproteinase 17; DKO, double KO; DMSO, dimethyl sulfoxide; FRMD8, FERM domain containing 8; GI, GI254023X; GW, GW280264X; HEK293T, human embryonic kidney 293T cell line; IP, immunoprecipitation.

Since mutating the iRhom2 RKR motif led to elevated basal ADAM17 activity, we asked whether this was associated with increased levels of mature ADAM17. To our surprise, mutation of this motif instead caused a significant reduction of mature ADAM17 level (Fig. 6*A*). This apparently paradoxical result led us to hypothesize that abnormally activated ADAM17 may trigger a homeostatic signal–attenuating degradation mechanism, in which the activated signaling protein is degraded to attenuate the signal, a relatively common theme in cell signaling (51). If this feedback mechanism exists, degradation of ADAM17 would be expected to be dependent on its own signal-generating catalytic activity. In support of this hypothesis, we found that inhibition of ADAM17 protease activity with a chemical inhibitor, GW280264X (which inhibits both ADAM17 and ADAM10), significantly rescued mature ADAM17 protein levels (Fig. 6*A*, see quantifications in Fig. 6*B*). A similar rescue was also seen with another inhibitor of ADAM17, BB94 (Fig. 6*A*). Importantly, a specific ADAM10 inhibitor, GI254023X, had no effect on ADAM17 levels (Fig. 6*A*), confirming that ADAM17 degradation depends specifically on its own activity. Consistent with a mechanism whereby ADAM17-dependent signaling leads to its own degradation from the cell surface, the lysosomal protein degradation inhibitor bafilomycin A1 also rescued the levels of mature ADAM17 in the presence of the activating iRhom2 RKR mutation (Fig. 6*A*, see quantifications in Fig. 6*B*). To summarize, mutating the iRhom2 RKR motif led to elevated ADAM17 basal activity, which in turn induced lysosomal-dependent ADAM17 degradation; this implies the existence of an iRhom2-dependent homeostatic feedback mechanism of ADAM17 activity.

We then asked whether the RKR motif (and the wider PRRKRM sequence) influences the binding of cytosolic interactors of iRhom2. The PRRKRM sequence contains a diarginine pair that represents a potential binding site for the coat protein complex I (COPI) that regulates ER vesicle trafficking (48, 52). However, we found that mutating the PRRKRM sequence did not alter the interaction between iRhom2 and beta-COP, a component of the COPI coatomer complex (Fig. 6*C*, see quantification in Fig. 6*D*). Diarginine signals have also been shown to bind to 14-3-3 proteins (53, 54), which we know to interact with the iRhom2 cytoplasmic domain (9, 10, 28); again, however, mutating the sequence had no detectable effect on 14-3-3 binding, at least for the isoform we tested (Fig. 6*C*, see quantification in Fig. 6*E*). Finally, despite the proximity of the PRRKRM sequence to the site of FRMD8 binding (22), the PRRKRM/As mutant iRhom2 also did not affect iRhom2–FRMD8 interaction (Fig. 6*F*, see quantification in Fig. 6*G*).

In contrast to the lack of effect on COPI, 14-3-3, or FRMD8, mutation of the RKR motif in iRhom2 greatly diminished its interaction with mature ADAM17 (Fig. 6*C*, see quantification in Fig. 6*H*). This reinforces the correlation between elevated ADAM17 activity and reduced iRhom2 binding, as previously observed upon stimulated activation of ADAM17 (9, 10). The reduced interaction between iRhom2 harboring the activating PRRKRM/As mutation and ADAM17 was not rescued by overexpression of FRMD8 (Fig. 6*F*), further supporting the conclusion that the RKR motif is unrelated to FRMD8 binding.

Although we have not yet discovered the mechanism by which the iRhom2 RKR motif regulates signaling, mutation of the RKR motif led to a decreased surface level of iRhom2 (Fig. 6*I*, see quantifications in Fig. 6*J*). As described previously, decreased iRhom2 at the cell surface level was observed upon ADAM17 activation by PMA (Fig. 3). This suggests that the RKR motif may regulate iRhom2 cell surface level, as well as affect the interaction between iRhom2 and mature ADAM17. By so doing, mutations in the RKR motif could modulate the inhibitory effect of iRhom2 on ADAM17, contributing to the elevated basal shedding activity of ADAM17.

## Discussion

The pivotal role of ADAM17 in regulating pathophysiological processes such as inflammation and growth factor responses has established it as a major target for both fundamental research and translational studies. The discovery of iRhoms as regulatory cofactors has provided key insights into the regulation of this multifunctional and essential sheddase and has positioned iRhoms as potential therapeutic targets. There has been significant progress in our mechanistic understanding, especially through the transmembrane and extracellular domains of the ADAM17–iRhom2 complex, especially when the complex structure was solved (7, 8, 9, 16). In contrast, the role of the iRhom2 cytoplasmic domain has been much less explored. In this study, we report the discovery of three distinct mechanisms by which the iRhom2 cytoplasmic domain interprets intracellular signals to regulate both stimulated and constitutive activity of ADAM17.

First, we have identified that the RSK2 serine/threonine kinase contributes to transducing cellular signals *via* the iRhom2 cytoplasmic domain. Indeed, RSK2 is necessary for the activation of the iRhom2–ADAM17 complex by oncogenic KRAS. RSK2 works in synergy with activated ERK to promote ADAM17 activation. Although the exact functional relationship between these two kinases and how they interact to promote ADAM17 activation remains unknown, we hypothesize that activated ERK may prime the iRhom2 cytoplasmic domain to allow RSK2 to function, a mechanism seen in other kinase regulatory networks. For example, polo-like kinase 1 requires CDK1-directed phosphorylation on polo-like kinase 1–binding scaffolds as a priming process (55). Note that, as well as RSK2 and ERK, KRAS itself was found to interact with iRhom2 (Fig. 1*B*), suggesting that the iRhom2 cytoplasmic domain may act as a scaffold or signaling hub, recruiting multiple components of the RAS–mitogen-activated protein kinase pathway.

The second mechanism we describe here is that cell surface levels of iRhom2 are downregulated upon PMA-stimulated ADAM17 activation, and, in parallel, that stabilization of iRhom2 at the cell surface, by overexpressing FRMD8, inhibits PMA-stimulated ADAM17 activity. Although we cannot rule out an indirect effect of FRMD8, we believe the negative correlation between iRhom2 cell surface level and ADAM17 activity suggests that iRhom2 may act to inhibit ADAM17 at or near the plasma membrane. This observation feeds into the narrative that iRhom2 is a multifunctional regulator, serving to modulate ADAM17 in distinct ways—positively and negatively—throughout their life cycles (7, 8, 9, 10, 16, 18, 19). The inhibition of ADAM17 activity by FRMD8 depends on the C terminus of FRMD8. In other FERM domain–containing proteins, like moesin and ezrin, the C terminus is responsible for F-actin binding (42). It would therefore be interesting in the future to ask whether the C terminus of FRMD8 is also responsible for F-actin binding and whether this may contribute to stabilizing iRhom2 at the cell surface.

Finally, we have identified an inhibitory motif in the iRhom2 cytoplasmic domain that controls ADAM17 activation. When this PPRKRM sequence (more specifically, RKR) is mutated, basal ADAM17 activity is abnormally elevated, leading to the consequent degradation of ADAM17 protein, probably through a homeostatic feedback loop. This inhibitory motif represents yet another mechanism by which iRhom2 regulates ADAM17, in this case helping to control the basal level of unstimulated ADAM17-dependent shedding. Since RKR mutants did not affect the recruitment of any known cytosolic interactors of iRhom2, employing a discovery proteomic approach to identify novel effectors would be valuable for uncovering the protein(s) that mediate this motif's inhibitory function. Interestingly, although the cytoplasmic domains of neither iRhom2 nor ADAM17 were resolved in the recent cryo-EM structure (16), AlphaFold2 predicts that the RKR motif in iRhom2 is positioned close to a tyrosine phosphorylation site (Y702) in the ADAM17 cytoplasmic domain when the two cytoplasmic domains are modeled together. This site is phosphorylated by Src kinase and contributes to the regulation of ADAM17 (56), presenting another avenue for future investigation.

Although iRhom1 and iRhom2 share similar core roles in regulating ADAM17 activity, albeit with different tissue specificities (13) and potentially distinct effects on ADAM17 substrate selectivity (18, 57), they differ markedly in their cytoplasmic domains, in contrast to their more conserved transmembrane and extracellular regions. This raises the possibility that iRhom1 and iRhom2 employ distinct regulatory mechanisms to control ADAM17 activity. Given the growing evidence that the iRhom2 cytoplasmic domain plays a critical role in regulating ADAM17 activity, including in the context of human disease (24, 25, 26, 27), understanding how these cytoplasmic differences contribute to the functional specificity of each iRhom protein will be an important direction for future research.

In conclusion, our findings establish the iRhom2 cytoplasmic domain as a central signaling hub of the iRhom2–ADAM17 sheddase complex. By uncovering multiple mechanisms through which intracellular signals modulate ADAM17 activity *via* this domain, we expand the current understanding of how multilayered regulation of ADAM17 activity is achieved by its cofactor, iRhom2. Importantly, these biochemical and functional insights help to fill a critical gap left by structural studies, which have so far been unable to resolve the cytoplasmic domains of the complex. Together, our work highlights the dynamic and versatile role of the iRhom2 cytoplasmic domain in regulating ADAM17, providing a framework for understanding how intracellular signaling intersects with proteolytic control in health and diseases.

## Experimental procedures

### Molecular cloning

pcDNA3.1(+) iRhom2 constructs were subcloned from the iRhom2 lentiviral construct used by Künzel *et al.* (22). Purified PCR products and digested vector were assembled using In-Fusion HD Cloning kit (Takara). pEGFP-KRAS G12V was the same as previously used by Boris *et al.* (21). The RSK2 gene was sourced from Sino Biological. Site-directed mutagenesis was performed to generate mutants of iRhom2 and RSK2 using the Cloned Pfu DNA polymerase AD (Agilent). For all constructs, single colonies were picked, and the extracted DNA was verified by Sanger sequencing (Source Bioscience).

### Cell culture and DNA transfection

Human embryonic kidney (HEK) 293T cells, HEK293T iRhom1/iRhom2 double KO cells, and HEK double KO cells stably expressing human iRhom2 wildtype (22) were cultured in high-glucose Dulbecco's modified Eagle's medium (Sigma–Aldrich) supplemented with 10% fetal bovine serum (FBS; Sigma–Aldrich), 2 mM l-glutamine, 100 U/ml penicillin, and 100 μg/ml streptomycin (all Gibco). Cells were cultured at 37 °C with 5% CO_2_ in a humidified cell culture incubator and were split twice a week using TrypLE Express (Gibco). All cell lines were tested mycoplasma negative.

Nonliposomal transfection reagent FuGENE HD (Promega) was used for transient DNA transfection in HEK293T cells 24 h after the cells were seeded according to the manufacturer's instructions. The ratio of DNA (microgram) and the transfection reagent (microliter) is 1:4, and OptiMEM (Gibco) was used to prepare the transfection mix.

### Coimmunoprecipitation

Cells were washed three times with ice-cold PBS before they were lysed in Triton X-100 lysis buffer (1% Triton X-100, 150 mM NaCl, 50 mM Tris–HCl, pH 7.5) supplemented with EDTA-free complete protease inhibitor mix (Roche) and 10 mM 1,10-phenanthroline. Cell lysates were centrifuged at 21,000*g* at 4 °C for 15 min, and the supernatants were used for coimmunoprecipitation. Immunoprecipitation was performed at 4 °C for at least 2 h with over-head rotation using anti-HA magnetic beads (Thermo Scientific). Beads were washed five times with Triton X-100 lysis buffer before the proteins were eluted by 2x sample buffer (0.25 M Tris–HCl [pH 6.8], 10% SDS, 50% glycerol, and 0.02% bromophenol blue) supplemented with 100 mM DTT. Samples were heated for 10 min at 65 °C and subjected to Western blotting analysis.

When samples were used for MS analysis, *n*-dodecyl β-d-maltoside radioimmunoprecipitation assay lysis buffer (1% *n*-dodecyl β-d-maltoside, 150 mM NaCl, 50 mM Tris–HCl, pH 7.5, 0.1% SDS, 0.5% sodium deoxycholate) supplemented with protease inhibitor cocktail, 10 mM 1,10-phenanthroline and PhosSTOP was used, and one extra wash with PBS in a new set of tubes was performed before MS sample processing.

### MS sample preparation and data analysis

Proteins pulled down by the HA-beads were processed for MS analysis using an on-beads digest protocol. Briefly, proteins were denatured with 4 M urea at room temperature for 10 min. The urea solution was diluted using freshly made 8 M urea solution in 50 mM triethylammonium bicarbonate buffer. Cysteines were reduced by incubating samples with 10 mM Tris (2-carboxyethyl) phosphine at room temperature for 30 min. To alkylate cysteines, samples were treated with 50 mM 2-chloroacetamide in dark at room temperature for 30 min. Protein samples were then predigested with endoproteinase LysC (Promega) for 2 h at 37 °C shaking, with a ratio of 1 μg of LysC for 100 μg of protein sample. Before trypsin digestion, urea was diluted down to 2 M into 50 mM triethylammonium bicarbonate buffer. CaCl_2_ was added to samples at 1 mM final concentration before samples proceeded to trypsin (Promega) digestion overnight at 37 °C shaking. The ratio of trypsin to protein sample is 1 μg trypsin for 40 μg of protein sample. The next day, use 1% TFA to stop the trypsin reaction and then centrifuge samples for 30 min at 14,000 rpm at 4 °C to remove aggregates. The collected supernatant was then subjected to a C18 desalt clean-up treatment using Oasis HLB1 Extraction cartridges (Waters). Samples were dried down using SpeedVac and stored at −20 °C. Dried peptides were resuspended into 2% acetonitrile/0.1% formic acid before LC–MS/MS analysis. Peptides were separated by nano-liquid chromatography (Thermo Scientific Easy-nLC 1000 or Ultimate RSLC 3000) coupled in line to a Q Exactive mass spectrometer equipped with an Easy-Spray source (ThermoFisher Scientific). Peptides were trapped onto a C18 PepMap100 precolumn (300 μm i.d. × 5 mm, 100 Å; ThermoFisher Scientific) using solvent A (0.1% formic acid, HPLC grade water). The peptides were further separated onto an Easy-Spray RSLC C18 column (75 μm i.d., 50 cm length; ThermoFisher Scientific) using a 60 min linear gradient (15–35% solvent B [0.1% formic acid in acetonitrile]) at a flow rate of 200 nl/min. The raw data were acquired on the mass spectrometer in a data-dependent acquisition mode. Full-scan MS spectra were acquired in the Orbitrap (scan range 350–1500 *m/z*, resolution 70,000; automatic gain control target, 3e6; and maximum injection time, 100 ms). The 10 most intense peaks were selected for higher-energy collision dissociation fragmentation at 30% of normalized collision energy. Higher-energy collision dissociation spectra were acquired in the Orbitrap at resolution 17,500, automatic gain control target 5e4, maximum injection time 120 ms with fixed mass at 180 *m/z*. Charge exclusion was selected for unassigned and 1+ ions. The dynamic exclusion was set to 20 s.

Tandem mass spectra were searched using Sequest HT in Proteome Discoverer software, version 1.4 (Thermo Fisher Scientific), against a protein sequence database containing 20,355 protein entries, including *Homo sapiens* proteins (UniProt release from 200,719) in which the iRhom2 protein sequence was replaced by the HA-tagged iRhom2 protein sequence and common laboratory contaminants. During database searching cysteines (C) were considered to be fully carbamidomethylated (+57,0215, statically added), methionine (M) to be fully oxidized (+15,9949, dynamically added), all N-terminal residues to be acetylated (+42,0106, dynamically added). Two missed cleavages were permitted. Peptide mass tolerance was set at 50 ppm on the precursor and 0.6 Da on the fragment ions. Data were filtered at a false discovery rate below 1% at the peptide-spectrum match (PSM) level. Label-free quantification was performed by normalized spectral abundance (NSAF) as previously described (58), as the number of spectral counts (PSM) that identify a protein divided by the protein length (L), the PSM/L value represents the SAF, which is then divided by the sum of PSM/L for all proteins in the experiment. NSAF values were calculated after common contaminants were removed. For better visualization of the data, NSAF values were multiplied by 100 (NSAF∙100). The statistical analysis was performed on NSAF values using a *t* test, and scatter plots were generated in Perseus (59). Proteins with a minimum fold change of two between each condition and a *p* value cutoff of 0.05 are highlighted on the plot.

### Concanavalin A enrichment

Concanavalin A (ConA) enrichment was performed to enrich glycosylated proteins and improve the separation of immature and mature ADAM17 forms in Western blot analysis. Cell lysates were prepared as described previously and incubated with 30 μl ConA sepharose (Sigma–Aldrich) at 4 °C for at least 3 h with over-head rotation. Beads were pelleted by centrifuging at 4000 rpm at 4 °C for 2 min and washed five times with Triton X-100 lysis buffer. Proteins were eluted with 2x LDS buffer (Invitrogen) supplemented with 50 mM DTT and 50% sucrose, followed by heating at 65 °C for 10 min.

### AP-shedding assay

Cells were seeded in a poly-l-lysine–coated 24-well plate in triplicate 24 h before transfection. About 150 ng AP-conjugated substrates were transfected with FuGENE HD transfection reagent. In KRAS-related experiments, 100 ng control plasmids or KRAS plasmids were transfected together with AP substrates. About 24 h after transfection, cells were washed with PBS and incubated in 300 μl colorless OptiMEM (Gibco; catalog no.: 11058-021) for 18 to 20 h. Then the supernatants were collected, and cells were lysed in 300 μl Triton X-100 lysis buffer supplemented with EDTA-free complete protease inhibitor mix (Roche) and 10 mM 1,10-phenanthroline. For stimulated shedding, cells were stimulated by 200 nM PMA in colorless OptiMEM. GW280264X (2 μM; Generon, AOB3632-5) or GI254023X (2 μM; Sigma–Aldrich, SML0789-5MG) was used to inhibit ADAM17 or ADAM10 activity. BI-D1870 (10 μM; Selleck Chemicals, S2843) was used to inhibit RSK activity. The supernatant (100 μl) and diluted cell lysates (100 μl) were independently incubated with 100 μl AP substrate p-nitrophenyl phosphate (Thermo Scientific) at room temperature, and the absorbance was measured at 405 nm with a plate reader (SpectraMax M5; Molecular Devices). The percentage of substrate release, that is, the ratio of released AP to the total AP (released in supernatant and unreleased in cell lysates), was calculated to minimize the effect of substrate level variance because of transfection efficiency.

### Western blotting

Proteins were separated by SDS-PAGE. For ConA-enriched samples, 4 to 12% Bis–Tris NuPAGE gradient gels (Invitrogen) and Mops running buffer (50 mM Mops, 50 mM Tris, 0.1% SDS, and 1 mM EDTA) were used. Otherwise, Novex 8 to 16% Tris–Glycine Mini Gels with WedgeWell format (Thermo Scientific) and Tris–Glycine running buffer (25 mM Tris, 192 mM glycine, and 0.1% SDS) were used. Proteins were then transferred to a methanol-activated polyvinylidene difluoride membrane (Millipore). Bis–Tris or Tris–glycine transfer buffer was used, respectively. Milk (5%) in PBS with Tween-20 was used for blocking and antibody incubation, and PBS with Tween-20 was used for washing. Enhanced chemiluminescence (ECL) reagent or ECL Select Western blotting detection reagent (both GE Healthcare) was used to detect antibody binding, and membranes were exposed to X-ray films (Thermo Scientific) or super-sensitive films (Amersham Hyperfilm ECL; GE Healthcare). The films were developed in an SRX-101A medical film processor (Konica).

The following antibodies were used: anti-ADAM17 (rabbit polyclonal, 1:2000 dilution; Abcam, ab39162), anti-beta-actin (mouse monoclonal, 1:2000 dilution; Santa Cruz, sc-47778), anti-beta-actin-horseradish peroxidase (HRP) (mouse monoclonal, 1:10,000 dilution; Sigma–Aldrich; A3854), anti-14-3-3 epsilon (rabbit polyclonal, 1:500 dilution; Cell Signaling Technology, 9635), anti-HA-HRP (rat monoclonal, clone 3F10, 1:2000 dilution; Roche, 12013819001), anti-RSK2 (rabbit polyclonal, 1:1000 dilution; Cell Signaling Technology, 9340), anti-V5-Tag (D3H8Q, rabbit monoclonal, 1:5000 dilution; Cell Signaling Technology, 13202), and anti-beta COP (mouse monoclonal, 1:1000 dilution; Sigma–Aldrich, G6160), anti-rabbit-HRP (goat polyclonal, 1:2500 dilution; Cell Signaling Technology, 7074), and anti-mouse-HRP (horse polyclonal, 1:2500 dilution, Cell Signaling Technology, 7076).

### Flow cytometry

For cell surface labeling, all procedures were performed on ice. Cells were washed three times with ice-cold PBS and gently detached from the plate in fluorescence-activated cell sorting (FACS) buffer (ice-cold PBS supplemented with 1% FBS). 1 × 10^6^ cells were centrifuged at 400*g* for 5 min at 4 °C. Antibodies were diluted in 100 μl FACS buffer (ice-cold PBS supplemented with 1% FBS). Cells were then resuspended in primary antibodies and incubated for 45 min and washed three times with FACS buffer with centrifugation of 400*g* for 5 min at 4 °C to pellet cells. Cells were then incubated with the fluorescent secondary antibodies for 30 min and washed three times with FACS buffer before the samples were resuspended in 400 μl FACS buffer and analyzed with a CytoFlex flow cytometer (Beckman). 4′,6-Diamidino-2-phenylindole was added immediately before the samples were loaded to the machine and incubated for 2 min. Cells stained only with the secondary antibody served as a control. Data were analyzed using the FlowJo software (BD Life Sciences).

The following antibodies were used: anti-ADAM17 (mouse monoclonal, 1:100 dilution; R&D Systems, MAB 9301), anti-HA-Tag (C29F4, rabbit monoclonal, 1:1000 dilution; Cell Signaling Technology, 3724S), anti-rabbit Alexa Fluor 647 (donkey polyclonal, 1:1000 dilution; Invitrogen, A-31573), and anti-mouse Alexa Fluor 488 (donkey polyclonal, 1:1000 dilution; Invitrogen, A-21202).

## Data availability

All data are contained in this article. Original images of blots are available on demand. The MS proteomics data have been deposited to the ProteomeXchange Consortium *via* the PRIDE (60) partner repository with the dataset identifier PXD063983. Supporting Excel file contains the reported protein and peptide data.

## Supporting information

This article contains supporting information (including figures and an Excel spreadsheet).

## Conflict of interest

The authors declare that they have no conflicts of interest with the contents of this article.
