# Supplementary material for: The cytoplasmic domain of the pseudoprotease iRhom2 mediates distinct signaling mechanisms to control activation of the cell surface protease ADAM17
Source: J Biol Chem. 2025 Aug 28;301(10):110643. doi: 10.1016/j.jbc.2025.110643 (PMC12516561; doi:10.1016/j.jbc.2025.110643)
Supplement: Supplementary legends [file mmc1.docx]

**Supplementary Figure Legends**

**The cytoplasmic domain of the pseudoprotease iRhom2 mediates distinct signaling mechanisms to control activation of the cell surface protease ADAM17**

Fangfang Lu^1^, Marjorie Fournier^2^, Matthew Freeman^1*^

^1^Sir William Dunn School of Pathology, University of Oxford, South Parks Road, Oxford OX1 3RE, UK

^2^Advanced Proteomics Facility, Department of Biochemistry, University of Oxford, Oxford, OX1 3QU, UK

**Figure S1** Control and collated data for experiments in Figure 2B-D. **A)** AP-shedding data from three independent experiments of Figure 2B, each dot represents the mean value of three transfectants of each experiment. Induction is calculated by dividing the shedding percentage of KRAS condition with that of GFP condition. **B)** AP-shedding data from three independent experiments of Figure 2C, each dot represents the mean value of three transfectants of each experiment. Induction is calculated by dividing the shedding percentage of KRAS condition with that of GFP condition. **C)** AP-shedding assay of iRhom1/2 DKO HEK293T cells co-transfected with WT or F307A iRhom2, GFP or oncogenic KRAS (G12V), as well as ADAM17 substrate AREG. n=3 transfectants, medium collected and assayed separately. **D)** AP-shedding data from three independent experiments of Figure 2D, each dot represents the mean value of three transfectants of each experiment. For A-D, Log₁₀-transformed data were statistically analyzed using ordinary one-way ANOVA, followed by Šídák's multiple comparisons test for pairwise comparisons as indicated. ns = not significant; ** = *p* < 0.01; **** = *p* < 0.0001. Adjusted *p*-values are shown. Error bars represent the standard deviation (SD).

**Figure S2** Control and collated data for experiments in Figure 4C. **(A)** AP-shedding data from three independent experiments of Figure 4C. **(B)** AP-shedding assay of EGF, ADAM10 substrate remained unaffected across different conditions. Data are from three independent experiments; each dot represents the mean value of three transfectants of each experiment. For A-B, Log₁₀-transformed data were statistically analyzed using ordinary one-way ANOVA, followed by Šídák's multiple comparisons test for pairwise comparisons as indicated. ns, not significant, * = *p* < 0.05, *** *p* = < 0.001. Adjusted *p*-values are shown. Error bars represent the standard deviation (SD).

**Figure S3** Repeats of experiments in Figure 5B and 5D. **(A-D)** AP-shedding assay of iRhom1/2 DKO HEK293T cells expressing EV (empty vector), WT or different mutant iRhom2. Cells were not stimulated, and medium was collected overnight. In each figure, n=3 transfectants, media collected and assayed separately. Log₁₀-transformed data were statistically analyzed using ordinary one-way ANOVA, followed by Dunnett’s multiple comparisons test against the WT condition. ns = not significant, * = *p* < 0.05, ** = *p* < 0.01, *** *p* = < 0.001. Adjusted *p*-values are shown. Error bars represent the standard deviation (SD).
