# Supplementary figures and images for: The cytoplasmic domain of the pseudoprotease iRhom2 mediates distinct signaling mechanisms to control activation of the cell surface protease ADAM17
Source: J Biol Chem. 2025 Aug 28;301(10):110643. doi: 10.1016/j.jbc.2025.110643 (PMC12516561; doi:10.1016/j.jbc.2025.110643)

**Figure S1****A**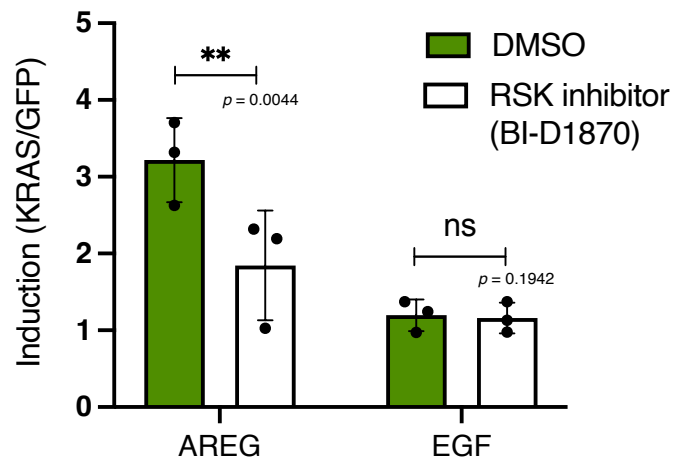**B**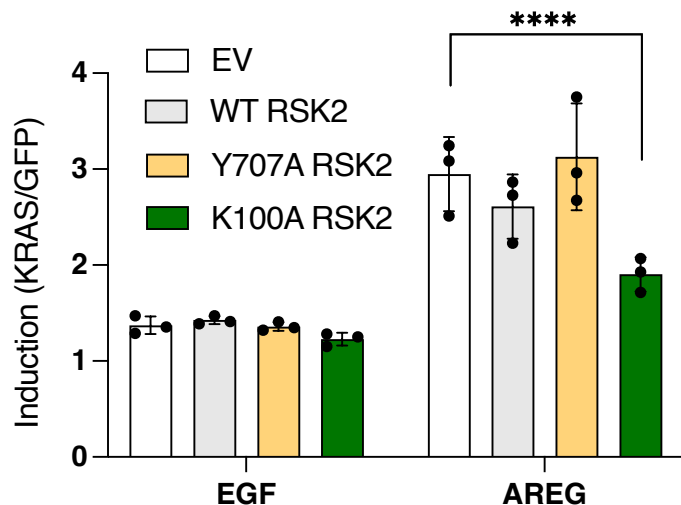**C**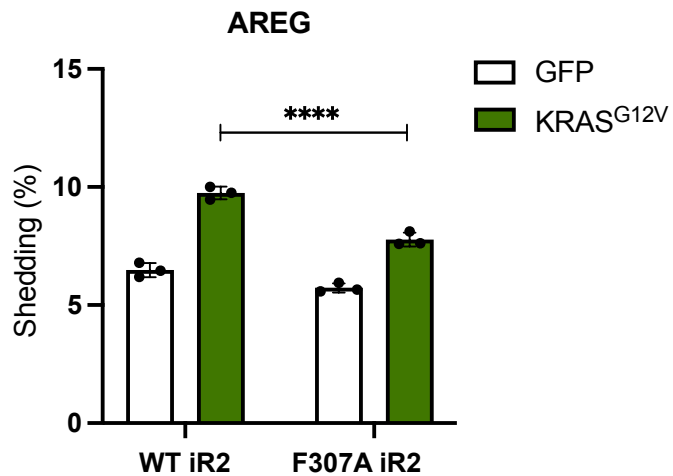**D**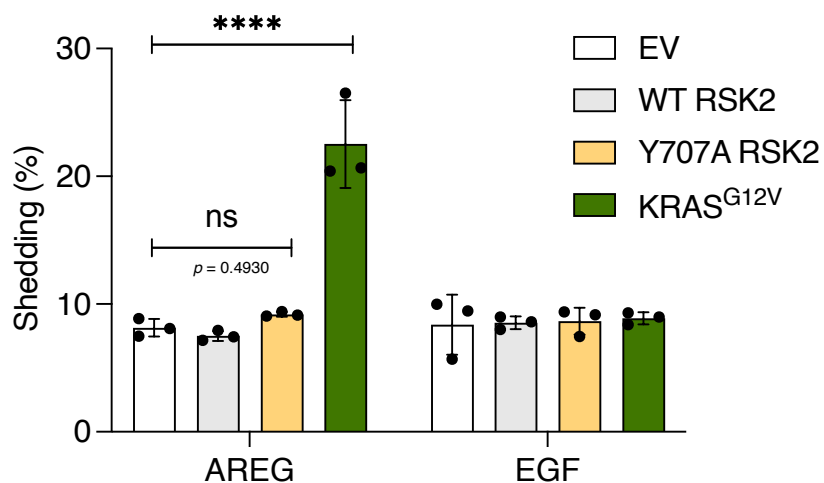

Supplement: Figure S2 [file mmc3.pdf]

**Figure S2**

**A**

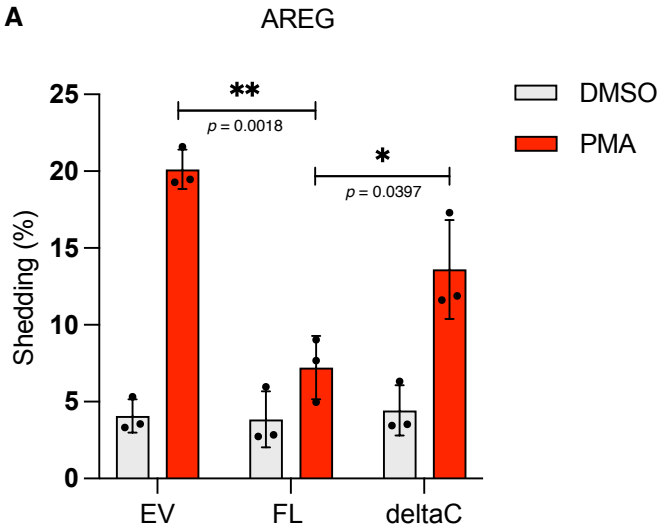

**B**

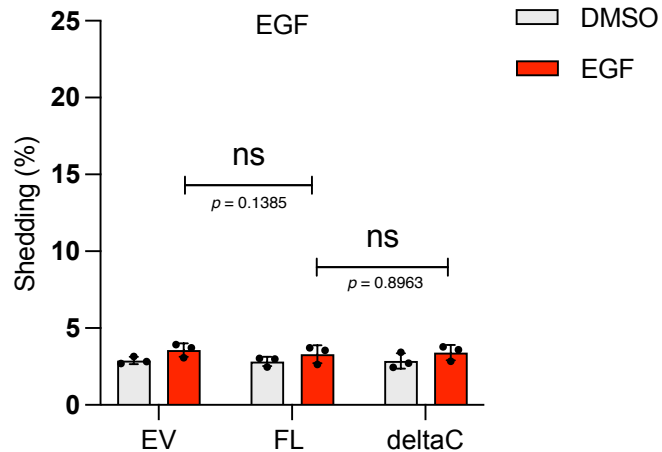

Supplement: Figure S3 [file mmc4.pdf]

**Figure S3****A**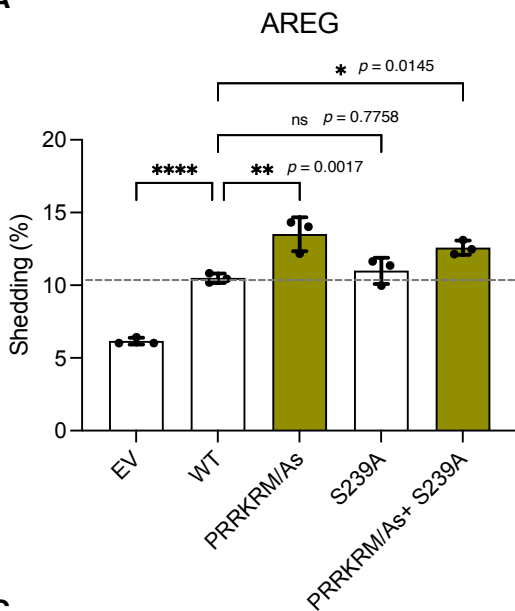**B**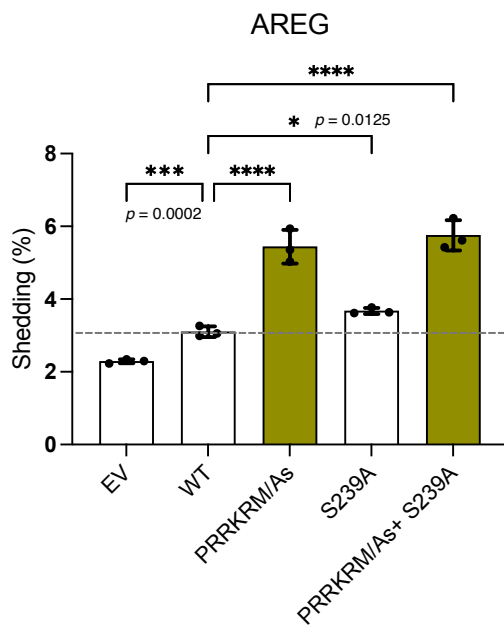**C**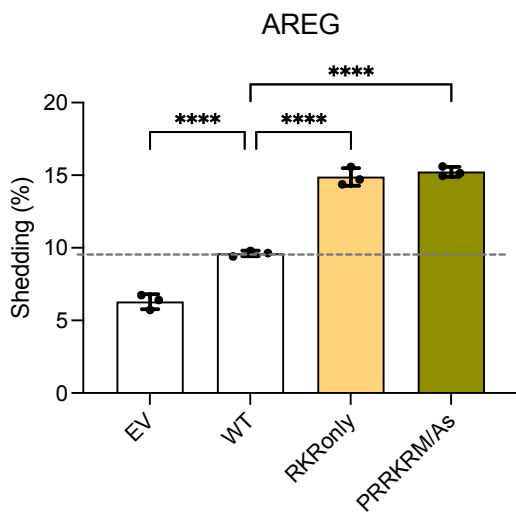**D**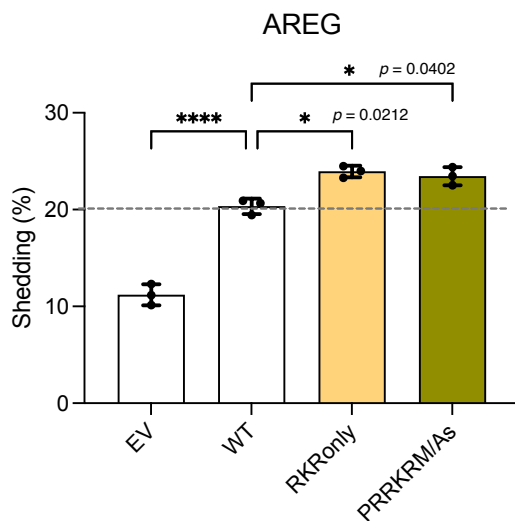

Supplement: Supporting information [file mmc5.pdf]
